# Influence of Development and Dietary Phospholipid Content and Composition on Intestinal Transcriptome of Atlantic Salmon (*Salmo salar*)

**DOI:** 10.1371/journal.pone.0140964

**Published:** 2015-10-21

**Authors:** Christian De Santis, John F. Taylor, Laura Martinez-Rubio, Sebastian Boltana, Douglas R. Tocher

**Affiliations:** Institute of Aquaculture, University of Stirling, Stirling FK9 4LA, Scotland, United Kingdom; University of Nordland, NORWAY

## Abstract

The inclusion of intact phospholipids in the diet is essential during larval development and can improve culture performance of many fish species. The effects of supplementation of dietary phospholipid from marine (krill) or plant (soy lecithin) sources were investigated in Atlantic salmon, *Salmo salar*. First feeding fry were fed diets containing either krill oil or soybean lecithin supplying phospholipid at 2.6%, 3.2%, 3.6% and 4.2% of diet. Fish were sampled at ~ 2.5 g (~1,990°day post fertilization, dpf) and ~10 g (2,850°dpf). By comparison of the intestinal transcriptome in specifically chosen contrasts, it was determined that by 2,850°dpf fish possessed a profile that resembled that of mature and differentiated intestinal cell types with a number of changes specific to glycerophospholipid metabolism. It was previously shown that intact phospholipids and particularly phosphatidylcholine are essential during larval development and that this requirement is associated with the inability of enterocytes in young fry to endogenously synthesize sufficient phospholipid for the efficient export of dietary lipid. In the immature phase (~1,990°dpf), the dietary phospholipid content as well as its class composition impacted on several biochemical and morphological parameters including growth, but these differences were not associated with differences in intestinal transcriptomes. The results of this study have made an important contribution to our understanding of the mechanisms associated with lipid transport and phospholipid biosynthesis in early life stages of fish.

## Introduction

The inclusion of intact phospholipids in the diet can improve culture performance of many fish species [1]. The beneficial effects of dietary phospholipids in fish include improved growth in both larvae and early juveniles, increased survival rates and decreased incidence of malformation in larvae, and perhaps increased stress resistance [1,2]. The requirement appears to be restricted to early life stages and no requirement has been established in adult fish of any species, although this is largely unstudied [3,4]. The phospholipid requirement can vary depending upon species and developmental stage (larvae or juveniles) from around 2% up to 12–14% of diet [5,6]. In the few studies where semi-purified phospholipid preparations have been investigated, the efficacy of individual phospholipid classes is commonly in the rank order phosphatidylcholine (PC) > phosphatidylinositol (PI) > phosphatidylethanolamine (PE), with PC generally being more associated with growth enhancement and PI more associated with survival and development [7–9].

In an early study in Atlantic salmon (*Salmo salar*), a dietary phospholipid requirement was demonstrated in fish up to 1.7 g initial weight, while in fish of 7.5 g initial weight dietary phospholipid did not enhance growth or survival [10]. In a recent study, we reinvestigated the phospholipid requirements of Atlantic salmon from first feeding through to smolt in fish fed diets containing either krill oil (a rich source of PC) or soybean lecithin (a source of PC and PI) ([11]). Survival and growth data confirmed that there was a requirement for dietary phospholipid supplementation in salmon fry at early life-stages up to 1990° day post fertilisation (dpf, ~ 2.5 g), and that the optimum level of phospholipid was around 2.6% when krill oil was used and 3.6% when using soy lecithin, with this difference possibly reflecting the level of PC delivered.

The mechanism underpinning the role of dietary phospholipids in early life stages of fish is not fully understood. Many studies established that the effects of dietary phospholipid were not dependent upon any physical characteristics that phospholipids may impart to feeds such as potentially enhanced emulsification and digestion of lipids [3]. Furthermore, the beneficial effects were not related to the delivery of important nutrients such as essential fatty acids, phosphate or the bases choline and inositol. For example, the growth enhancement in salmon fry elicited by dietary soybean lecithin was not replicated by choline supplementation [10,12]. However, several studies reported that feeding diets deficient in phospholipid resulted in intestinal steatosis in fish larvae with accumulation of lipid vacuoles or droplets in enterocytes, which led to the theory that dietary phospholipids may be required for the efficient export of dietary lipid from the intestine [13–17]. As phospholipid is required for lipoprotein assembly, it was further proposed that the growth enhancing effects of dietary phospholipid were due to early life stages of fish having limited ability for *de novo* phospholipid biosynthesis [13,18,19]. Thus, dietary phospholipid would increase the efficiency of transport of dietary fatty acids and lipids from the intestine to the liver and, ultimately, the rest of the body through enhanced lipoprotein synthesis [3]. Therefore, this suggested that the molecular mechanism underpinning phospholipid requirement would be located specifically in intestinal tissue/cells. Our recent study in Atlantic salmon provided evidence that intestinal steatosis could develop in fish of up to 1990°dpf (~2.5 g) when fed a low (unsupplemented) phospholipid diet. Although only 20% of fish fed the unsupplemented diet developed steatosis (two out of ten fish) this was abolished in fish fed 2.6% krill phospholipid and 3.6% soy lecithin [11].

The aim of the present study was to determine the molecular mechanism(s) underpinning the requirement for dietary intact phospholipid in early life stages of Atlantic salmon. Specifically, we sought to determine the effects of development and phospholipid supplementation on the intestinal transcriptome. Atlantic salmon fry were fed diets containing either krill oil or soybean lecithin supplying phospholipid at 2.6%, 3.2%, 3.6% and 4.2% of diet from first feeding through to parr-smolt transformation. Fish were sampled at ~ 2.5 g (1990°dpf) and ~10 g (2850°dpf). Comparisons of intestinal transcriptomes were specifically chosen to elucidate and discriminate effects of development, dietary phospholipid content, and phospholipid class.

## Methods and Materials

### Dietary trial and feeds

The feeding trial was carried out at the University of Stirling freshwater facilities with all experimental procedures conducted in compliance with the Animals Scientific Procedures Act 1986 (Home Office Code of Practice. HMSO: London January 1997) under project licence PPL70/7916 “Environmental Regulation of Fish Physiology” and personal licence number 177395F09 (J.F. Taylor) in accordance with EU regulation (EC Directive 86/609/EEC) and approved by the Animal Ethics and Welfare Committee of the University of Stirling. Mortality was very low (3.5 to 5.5%, with no difference between treatments) and entirely within normal expected levels for these nutritional studies classified as “mild”. There were no unexpected mortalities during the experiment and fish were monitored at least twice daily and any fish showing clinical signs of disease as defined under the licence conditions were euthanized by an overdose of anaesthetic (MS222, PHARMAQ, UK). All sampled fish were euthanized by the same method. Full details of the trial were provided previously [11]. Briefly, Atlantic salmon were fed from first feeding to smolt on diets containing increasing phospholipid content. Other than phospholipid, diets were formulated to meet the dietary requirements of salmon [20] and manufactured by BioMar AS (Tech Centre, Brande, Denmark). A basal diet containing 1.5% phospholipid (diet B) was supplemented with either krill oil or soybean lecithin to produce feeds with 2.6, 3.2, 3.6 and 4.2% total phospholipid (diets S2.6, S3.2, S3.6 and S4.2 when supplemented with soybean lecithin, and K2.6, K3.2, K3.6 and K4.2 when supplemented with krill oil). Full details of phospholipid preparations, diet formulations and compositions and trial design are given in Taylor et al [11].

### Intestine samples and RNA extraction

Environmental parameters were recorded twice daily for the duration of the trial. Fish were sampled for growth at approximately 1 g (1400°dpf), 2.5 g (1990°dpf), 5 g (2350°dpf), 10 g (2850°dpf), 30 g (3250°dpf) and smolt (3800°dpf). Fish were also sampled at these time points for histological examination of intestine and full details of the sampling protocols and analytical procedures were given previously [11]. In addition, the entire intestinal tract was collected from six fish per tank (12 per dietary treatment) and immediately placed in 1 ml of RNALater (Sigma-Aldrich, Dorset, UK) and processed as per manufacturer’s instructions. Total RNA was extracted from individual intestinal samples using TRI Reagent essentially according to manufacturer’s instructions (Sigma-Aldrich). RNA quantity, integrity and purity were assessed by agarose gel electrophoresis and spectrophotometry (NanoDrop ND-1000, Thermo Scientific, Wilmington, USA).

### Transcriptome analysis

#### Transcriptomic comparisons

The specific developmental stage and dietary treatments for comparison were carefully chosen to enable the analyses to discriminate the effects of fish development, dietary phospholipid content, and phospholipid class. Thus, the intestinal transcriptome of fry fed diet B at 1990°dpf (~ 2.5 g) was compared with intestinal transcriptomes from parr fed diet B at 2850°dpf (~ 10 g) to determine the effects of development, and with fry (1990°dpf) fed S2.6 and S3.6 (dietary phospholipid content) and S2.6 and K2.6 (phospholipid class) to determine the effects of dietary phospholipid supplementation. The choice of dietary phospholipid supplementation to be further analysed and individual contrasts was made based on evidence of improved growth and reduced steatosis (see summary in section 3.1) observed in our previous study [11].

#### Microarray hybridization protocol

Transcriptome analysis was conducted using a custom-made 4 x 44K Atlantic salmon oligo microarray (Agilent Technologies, Wokingham, UK; ArrayExpress accession no. A-MEXP-2065) described in detail previously [21]. The salmon custom array and laboratory procedures utilized have been used widely and validated in several previous studies [21–27]. The full laboratory protocol and pipeline for bioinformatics analyses are reported in detail in De Santis et al. [27]. Briefly, RNA was extracted from whole intestines pooled from two fish from the same tank using TRI Reagent (Sigma-Aldrich, Dorset, UK) and analysed as a single biological replicate, which provided 3 replicates per tank and 6 per dietary treatment. The RNA samples were amplified using TargetAmp^™^ 1-Round Aminoallyl-aRNA Amplification Kit, (Epicentre Technologies Corporation, Madison, Wisconsin, USA) following manufacturer recommended procedures. Aminoallyl-amplified RNA (aRNA) samples were labelled with Cy3 dye (GE HealthCare Life Sciences, Buckinghamshire, UK) while a pool of all aRNA samples was labelled with Cy5 dye (GE HealthCare Life Sciences) and used as a common reference. A dual-label common reference design was adopted, where equal amounts of each individual aRNA sample and the common reference pool were competitively hybridized to one array. Throughout the experiment samples were randomized to avoid samples from the same treatment being overrepresented in a particular batch in order to prevent unintentional biases. Details of the microarray experiment were submitted to ArrayExpress under accession number E-MTAB-3673.

#### Data pre-processing and differential expression

Data analysis was performed using R v.3.0.1 and Bioconductor v.2.13 [28,29]. Quality control, data pre-processing and identification of differentially expressed features/genes were conducted using the package limma [30]. Quality filtering and control was performed as described previously [27]. Multiple testing correction (False Discovery Rate, FDR) was used for differential expression analysis [31] and, unless otherwise specified, *q* < 0.05 was used as cutoff, where *q* represents *p* value corrected for FDR. Features of the array were annotated using BLAST 2.2.29+ (blastx) against the entire non-redundant protein database as well as using the KEGG Automatic Annotation Server to obtain functional annotations [32,33]. A total of 89.6% of all probes were returned with a BLAST annotation (annotation date Dec 2014) with e-value < 0.001, while 59% of probes were returned with a functional annotation (KEGG identifier) using the KAAS server. Features representing the same target gene implied from KEGG annotation were reduced into a unique value obtained by selecting the feature with the highest F-value across all contrasts. A new dataset was therefore generated for further analyses where each gene was represented by one feature only. Merging features resulted in a dataset of 6824 annotated features each targeting a unique gene.

#### Data mining

*Overview of differential expression*. All figures plotting differentially expressed genes were produced using the R package *ggplot2* [34]. For figures involving functional information, the KEGG database was used as the preferred classification system.

*Gene-Set Enrichment Analysis (GSEA)*. Unique annotated sequences were analyzed using the R function gage of the software package *gage* (Generally Applicable Gene-set Enrichment, [35]) to identify mechanistic changes as suggested by coordinated expression changes in gene-sets. One-direction (*1d*) test was performed. Gene-sets with a *q* < 0.05 were considered significant. KEGG classification was used for these analyses and all figures were produced using the software package *ggplot2*.

### Identification and validation of candidate genes for molecular phenotyping

Reverse transcriptase RT-qPCR was used to validate the microarray results and to test a number of candidate genes in all treatments including those not analyzed by microarray (100 g kg^-1^ and 200 g kg^-1^). Results were consistent between microarray and RT-qPCR and are provided as S1 Table. Complete methodology, primer design and results of individual gene expression analysed by RT-qPCR were published separately [36].

## Results

### Growth and intestinal histology

Full results of the dietary trial are given in Taylor et al. [11], but are summarised here to provide context for the samples investigated in the present study. Highest growth was obtained in fish fed 2.6% krill phospholipid and 3.6% soybean phospholipid. There was a clear difference in the pattern of growth observed between fish early in development up to 2.5 g (1990°dpf) with SGR (0–1990°dpf) showing significantly better growth performance in fish fed K2.6 and S3.6 compared to fish fed the basal diet B, whereas there was no difference in SGR between dietary treatments after 2.5 g to smolt (1990–3800°dpf). Diets K2.6 and S3.6 were characterised by having similar PC contents. Intestinal steatosis (assessed in the mid intestine) was observed at 2.5 g in fish fed both the B (20% of fish affected) and S2.6 (10% of fish affected) diets, but no steatosis was observed in intestine of fish fed any of the krill phospholipid-supplemented diets or in fish fed S3.2 or higher levels of soybean phospholipid. In addition, no steatosis was observed in the intestines of fish fed any of the dietary treatments at 10 g or higher.

### Transcriptome analysis overview

The analysis of differential expression revealed that most of the changes observed were associated with developmental stage (Table 1). Thus, the comparison between fry (2.5 g) and parr (10g) fed diet B showed 2446 differentially expressed genes (*q* < 0.05). In contrast, dietary effects were minimal using this stringency of cutoff and no differences were observed with only 2 genes showing significantly different expression in the comparison B—S3.6. To avoid being over conservative for the dietary contrasts, we adopted a reduced stringency cutoff and selected genes that had a *p* (uncorrected) < 0.005 and absolute fold change > 1.3. With this cut-off a larger number of genes were identified (Table 1), however these results were interpreted cautiously and only considered biologically relevant when affected by different dietary treatments simultaneously (see following sections).

**Table 1:** Number of differentially expressed genes between intestinal transcriptomes of fish at 1990°dpf fed the basal, unsupplemented diet (B, 2.5g) and fish fed the other treatments selected to enable effects of development (B, 10g), phospholipid content (S2.6 and S3.6) and phospholipid class (K2.6 and S2.6) to be determined.

Contrast	*q* < 0.05 (BH)	FC > 1.3;*p* < 0.005
B 2.5g vs B 10g	2446	1699
B 2.5g vs K2.6 2.5g	0	60
B 2.5g vs S2.6 2.5g	0	68
B 2.5g vs S3.6 2.5g	2	36

Fish were 1990°dpf (~2.5g) and 2850°dpf (~ 10g)

BH indicates Benjamini and Hochberg correction.

### Developmental changes of intestinal gene expression

#### De novo phospholipid biosynthesis pathway

**Fig 1:**
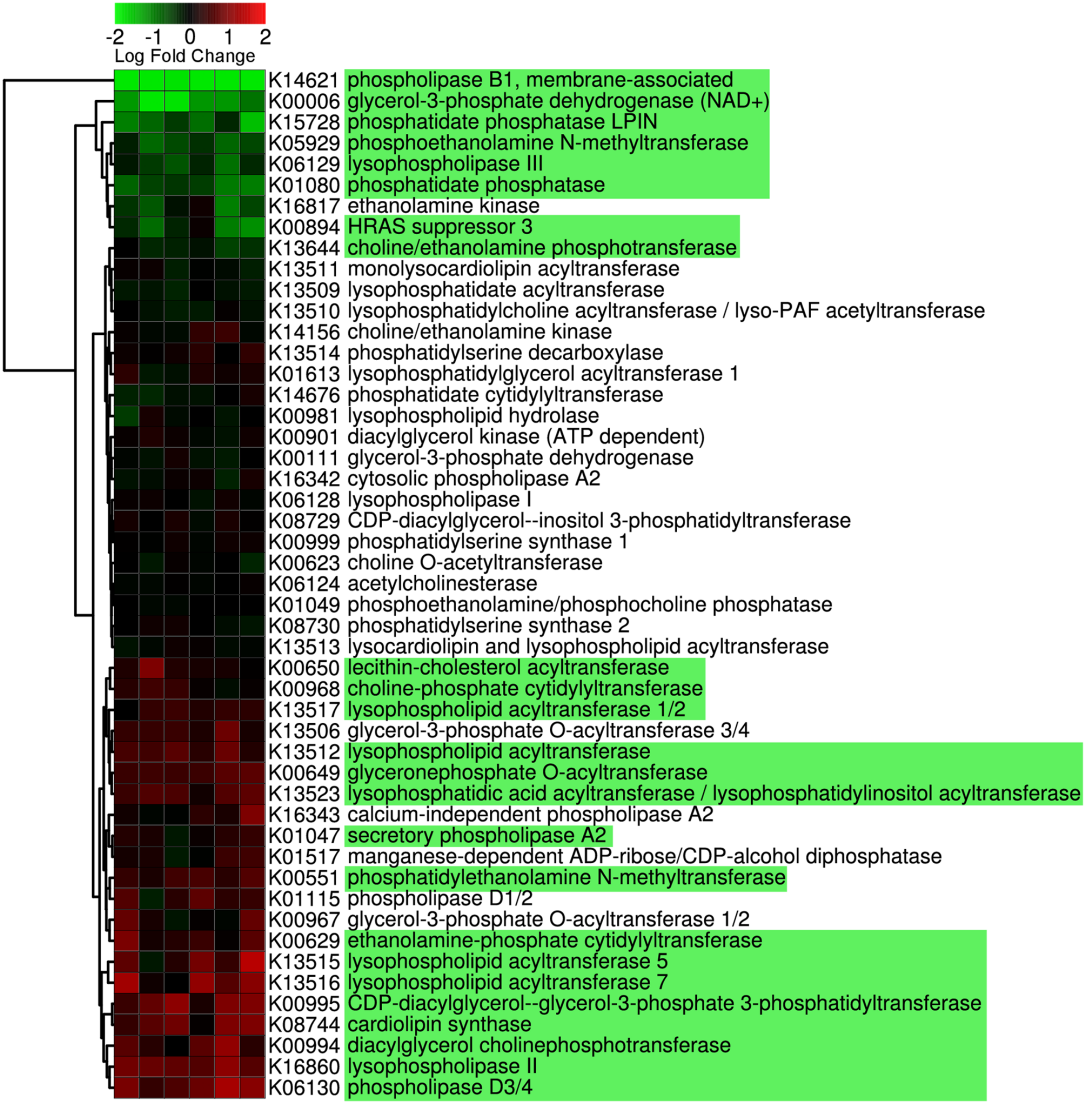
Genes participating in glycerophospholipid metabolism (Kegg ID ko00564). Values represent expression in the intestine of 10 g parr compared to 2.5 g fry fed the basal unsupplemented (low phospholipid) diet and are presented for each replicate independently. Genes highlighted in green are statistically significant (*q* < 0.05). Kegg identifiers (Kxxxxx) are specified for each gene. Data were plotted using heatmap.2 [37] and rows were clustered according to Euclidean distance.

Glycerophospholipid metabolism was significantly altered in the intestine of parr (10 g) compared to that of fry (2.5 g) (Fig 1). A total of 16 genes were significantly up-regulated and 8 were down-regulated (*q* < 0.05). Amongst up-regulated genes there were both genes coding for enzymes with phospholipolytic activity such as several phospholipases (D3/4, A2, lysophospholipase 2) as well as others with biosynthetic functions including a number of specific acytransferase enzymes. Genes involved in the biosynthesis of phosphatidylcholine using choline increased in 10 g fish compared with 2.5 g, including diacylglycerol cholinephosphotransferase and lecithin-cholesterol acytransferase. A gene coding for a second enzyme with phosphotransferase activity (choline/ethanolamine) was slightly down-regulated, however it is noteworthy that the expression of this gene was the only deviation detected between RT-qPCR and microarray results and therefore this result should be interpreted cautiously. Altered expression of other lipogenic enzymes was also observed, including lysophospholipid acyltransferase and cardiolipin synthase. The down-regulated genes included two phospholipases (B1 and lysophospholipase III), two phosphatidate phosphatases, and glycerol-3-phosphate dehydrogenase (Fig 1).

#### Global changes of gene expression (GSEA)

**Fig 2:**
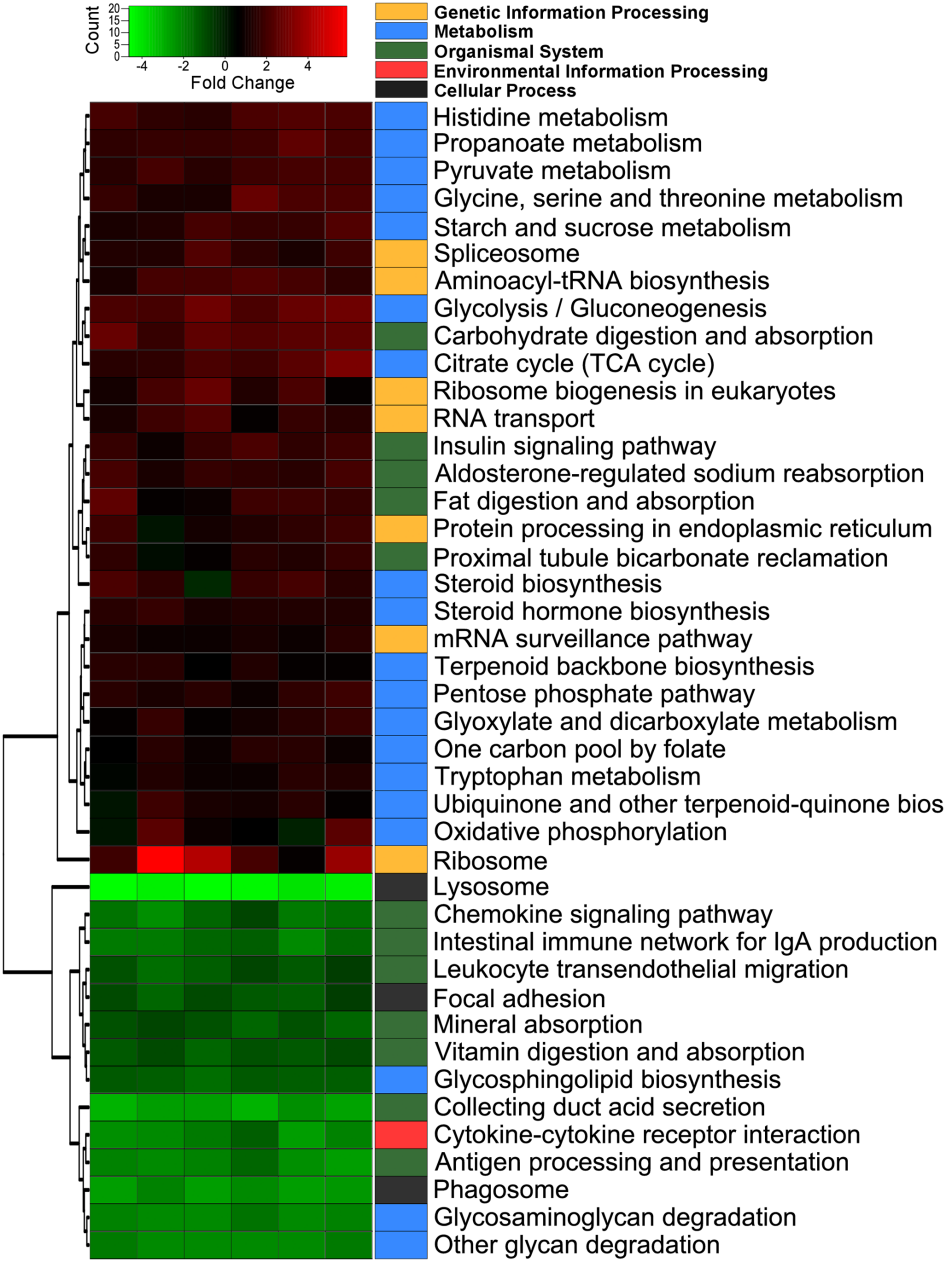
Heatmap plotting GAGE results from the *1d* test in the intestine of 10 g parr compared to 2.5 g fry fed the basal unsupplemented (low phospholipid) diet. All gene-sets with *q* < 0.05 are shown. Pathways are colour-coded based on the broad classification category (Kegg). Further details are provided in S2 Table. Data were plotted using heatmap.2 [37] and rows were clustered according to Euclidean distance.

The output of GSEA describing changes occurring in the intestine during development from 2.5 g fry to 10 g parr indicated that transcriptional changes could be summarized by 42 differentially expressed gene-sets (*q* < 0.05), 14 of which were down-regulated and 28 up-regulated (Fig 2). Among the most significant down-regulated gene-sets were those related to the lysosome and phagosome, along with pathways involved in immune response such as antigen processing and presentation, intestinal immune network for IgA production, cytokine-cytokine receptor interaction and chemokine signalling pathway. Among up-regulated gene-sets, there was an over-representation of metabolic pathways and genes controlling the processing of genetic information. Notably, a highly significant increase of glycolysis, citrate cycle, glycine-serine-threonine metabolism and histidine metabolism was observed in the intestine of 10 g parr compared with that of 2.5 g fry. To a lesser extent, other metabolic pathways also increased in parr including biosynthesis of steroids, pyruvate metabolism and pentose phosphate pathways, among others. Furthermore, the expression of protein processing gene-sets also increased including ribosome, aminoacyl-tRNA biosynthesis, RNA transport, ribosome biogenesis and protein processing in the endoplasmic reticulum. Finally, increased expression of genes involved in the digestion and absorption of lipids and carbohydrates was detected, whereas those involved in digestion and absorption of vitamins and minerals were slightly decreased.

### Transcriptional response to dietary phospholipid content and class

#### De novo phospholipid biosynthesis pathway

**Fig 3:**
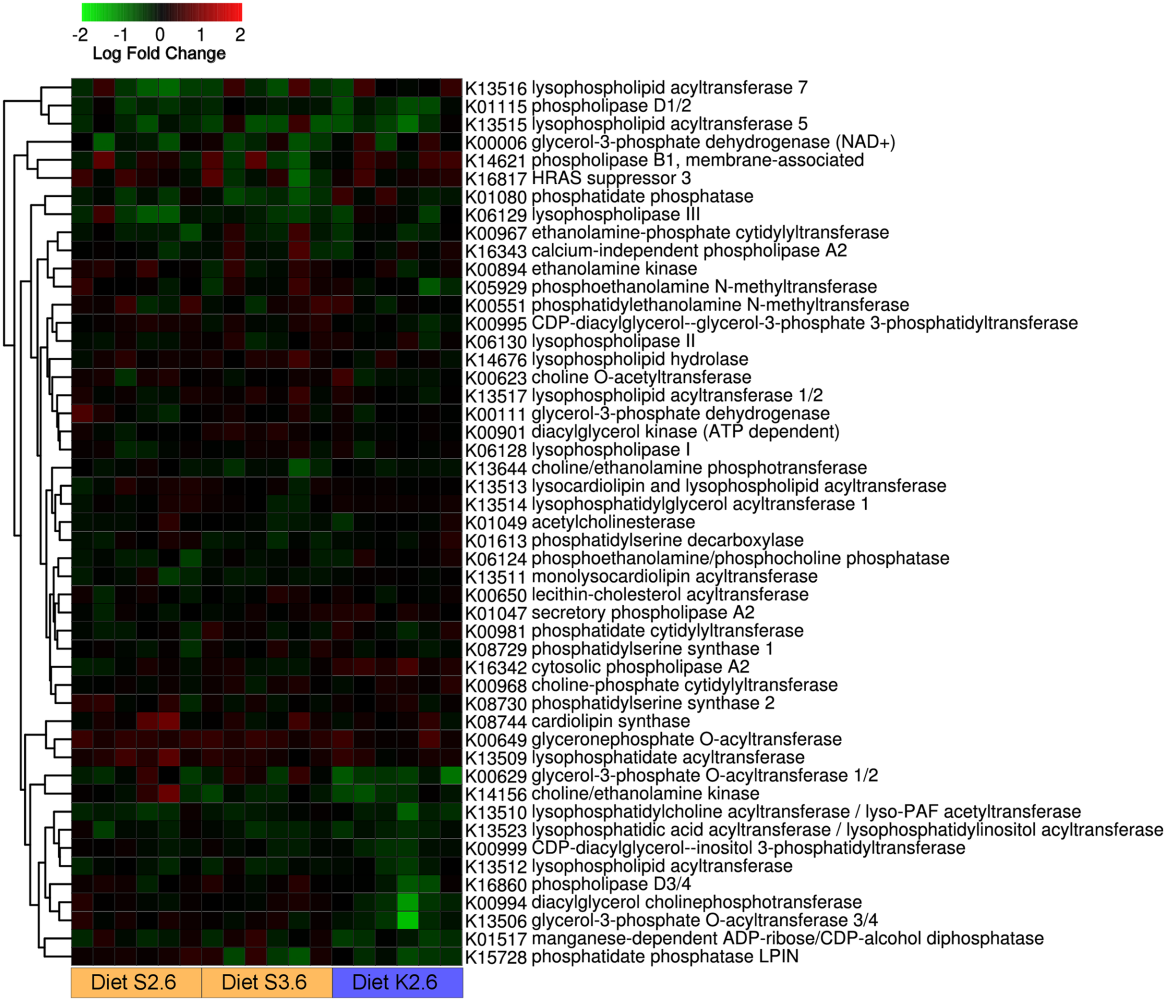
Genes participating to the glycerophospholipid metabolism (Kegg ID ko00564). Values represent expression in the intestine of 2.5 g fish fed diet S2.6, S3.6 and K2.6. Expression values are reported for each individual replicate compared to average of 1.5g fish fed the basal unsupplemented diet (1.5% phospholipid content). Kegg identifiers (Kxxxxx) are specified for each gene. Data were plotted using heatmap.2 [37] and rows were clustered according to Euclidean distance.

In contrast to development, dietary treatment did not significantly affect any genes of glycerophospholipid metabolism in the intestine of 2.5 g fry (Fig 3). Expression of two groups of genes presented a trend, albeit not statistically significant. The first included glyceronephosphate (dihydroxyacetone phosphate) O-acyltransferase and lysophosphatidate acyltransferase, both up-regulated by the inclusion of soy lecithin (S2.6 and S3.6) in the diet. The second was affected by inclusion of krill oil (K2.6) and included several genes involved in phosphatidylcholine biosynthesis such as choline-ethanolamine kinase, lysophosphatidylcholine acyltransferase, lysophospholipid acyltransferase, and diacylglycerol cholinephosphotransferase that were down-regulated.

#### Global changes of gene expression (GSEA)

**Fig 4:**
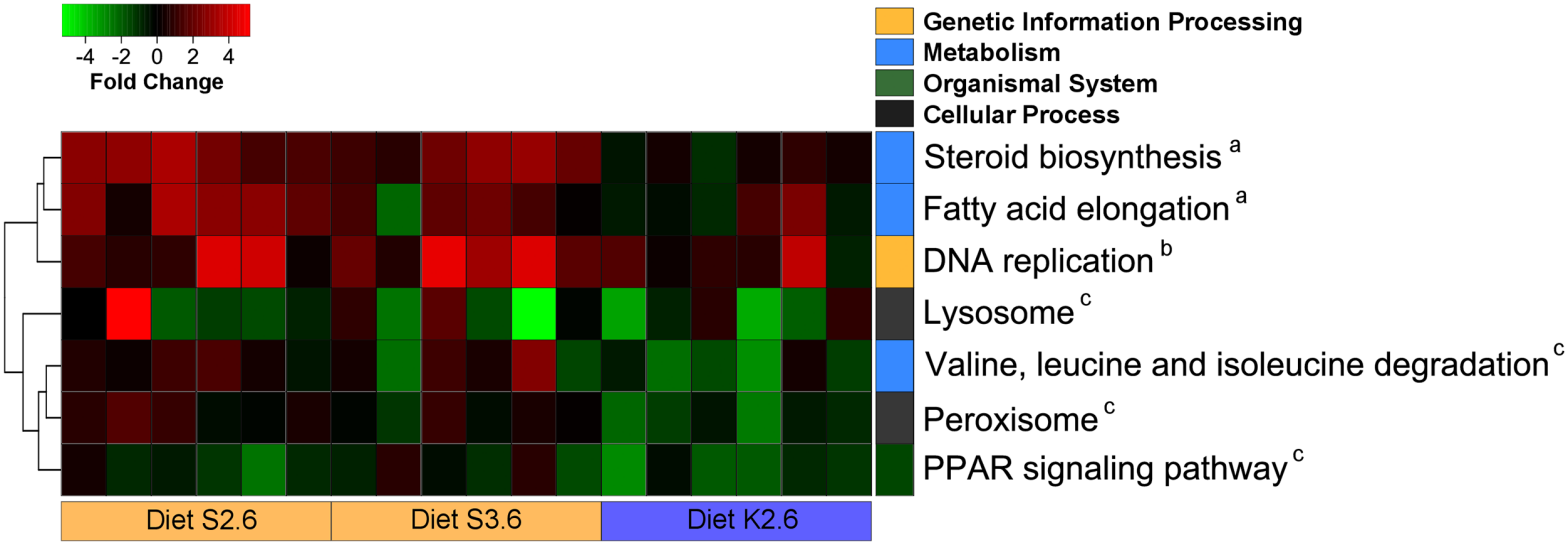
Heatmap plotting GAGE results from the *1d* test in the intestine of 2.5 g fry fed diets S2.6, S3.6 and K2.6. Expression values are reported for each individual replicate (n = 6) compared to.fry (2.5g) fed the basal unsupplemented (low phospholipid) diet. Only gene-sets with *q* < 0.05 are shown. Pathways are colour-coded based on the broad classification category (Kegg). Further details are provided in S2 Table. Gene-sets marked with an “a” are differentially expressed in S2.6 compared to B, “b” in S3.6 vs B and “c” in K2.6 vs B. Data were plotted using heatmap.2 [37] and rows were clustered according to Euclidean distance.

Consistent with the individual gene statistics, GSEA did not detect any major processes affected by dietary supplementation with phospholipid. Among processes considered statistically different there was up-regulation of steroid biosynthesis and fatty acid elongation in fish fed S2.6 compared to diet B. Although these lipogenic pathways were not significantly different in S3.6-fed fish a similar trend was observed (Fig 4). Diet S.3.6 significantly induced up-regulation of the DNA replication pathway. Four gene-sets were down-regulated when fish were fed K2.6, compared to diet B, including lysosome and peroxisome, valine-leucine and isoleucine degradation and PPAR signalling pathway. Regardless of the above, it should be noted that the *q* values resulting from GSEA of dietary treatments were generally only marginally significant (i.e. 0.01 < *q* < 0.05) as opposed to the developmental changes that were highly significant (i.e. *q* < 0.00001) (S2 Table).

#### Common effects of krill and soy phospholipid

To understand common mechanisms affected when phospholipid requirement was met, we examined the genes differentially expressed in fry fed both S3.6 and K2.6 compared with the control treatment B. These two treatments resulted in similar growth rate and were both effective in reducing steatosis to baseline levels. Five genes were found differentially expressed in both treatments and they were similarly affected in terms of expression. These genes included apolipoprotein A-IV, ADP-ribosylation factor 1 and glutamine synthetase, all down regulated in both S3.6 and K2.6 compared to the control, whereas the cytosolic sulfotransferase 3 and receptor-type tyrosine-protein phosphatase kappa were up-regulated (Table 2).

**Table 2:** Genes differentially expressed (p < 0.005; absolute fold change > 1.3) in both S3.6 and K2.6 compared with the control diet B.

			*S3*.*6 vs B*		*K2*.*6 vs B*	
Probe Name	KOID	KEGG_ProteinName	FC	*p* Value	FC	*p* Value
Ssa#S31963680	K08760	Apolipoprotein A-IV	-1.97	0.0003	-1.97	0.0003
Ssa#S35588230	K07937	ADP-ribosylation factor 1	-1.54	0.0006	-1.75	< 0.0001
Ssa#STIR02816	K01025	Cytosolic sulfotransferase 3	1.73	< 0.0001	1.51	0.0004
Ssa#STIR21285	K01915	Glutamine synthetase	-1.75	0.0002	-1.68	0.0004
Ssa#STIR24718	K06776	Receptor-type tyrosine-protein phosphatase kappa	1.32	0.0009	1.31	0.0011

*p* Value is the FDR-uncorrected *p* value

FC is fold change, positive values denote up-regulation compared to control diet B and vice versa

## Discussion

The objective of the present study was to elucidate transcriptional changes underlying the mechanisms of phospholipid requirement during early life stages of Atlantic salmon. Specifically, the effects of development and dietary phospholipid supplementation (soybean lecithin or krill oil) on the intestinal transcriptome were determined. Our results indicated that the intestine of salmon undergoes a dramatic restructuring at a transcriptional level between 1990°dpf (~ 2.5 g) and 2850°dpf (~ 10 g). This restructuring included, among others, genes involved in glycerophospholipid biosynthesis. Critically, although dietary supplementation of sources with different phospholipid composition (e.g. PC, PE and PI) such as soy lecithin and krill differentially influenced growth, deformities and intestinal steatosis of fry up to 2.5 g (intestinal immaturity), microarray analysis did not detect substantial alteration of the intestinal transcriptome.

Many fish species are known to require dietary intact phospholipids at early developmental stages [3,4]. The dietary phospholipid requirement for Atlantic salmon was recently reported to be around 2–3% depending upon source [11], marginally lower than that (4%) previously reported by Poston [10,12]. Taylor et al. [11] showed that salmon fry up to 1990°dpf were susceptible to developing intestinal steatosis (20% of fish affected) if the minimum level of dietary phospholipids was not met, an observation reported previously for other fish species such as carp [13]. These observations led to the hypothesis that intact phospholipids were required for the formation of lipoproteins such as the chylomicrons involved in packaging and transport of dietary lipid from the intestine to other tissues through the circulatory system [3]. In support of this hypothesis, it was demonstrated that intestinal steatosis, although only present in 20% of fish assessed, was abolished either by increasing the level of dietary phospholipid or as the intestine developed with time [11]. The mechanisms underlying the maturation of the intestinal tract, including the transition from being dependent on dietary intact phospholipid to being independent and, presumably, having the ability for endogenous production in the intestine are largely unknown.

In vertebrates, maturation of the intestine is tightly regulated at transcriptional and proteomic level [38–40]. Data from mammalian systems suggests that maturation is determined by the progression of proliferating cells into differentiated cells such as for example the case of the crypt-villi axis [39,40]. Proliferating and differentiated cells are characterized by distinctive transcriptional profiles, with the former displaying a higher expression of genes involved in pathways leading to RNA processing and protein synthesis, and the latter showing profiles reflecting increased potential for brush border assembly and nutrient uptake as well as other metabolic functions (e.g. glycolysis). In fish, it is known that the digestive tract undergoes substantial remodelling and maturation throughout development in early life stages and that this does not occur gradually but in spurts [41,42]. The morphological and functional development of salmon gut from 7 to 144 days post hatching (dph) was recently described [43]. Histology showed GI tract segmentation was evident from 54 dph and qPCR performed on whole intestine showed gene expression increased from first feeding [43]. In the present study we investigated older fish, between 162 and 225 dph (1990 and 2850°dpf), and the results suggested that the intestine underwent further maturation between these two stages.

At the molecular level, this was supported by extensive alterations of the intestinal transcriptome. Transcriptional changes included the up-regulation of gene-sets involved in digestion and absorption of lipid and carbohydrate as well as a number of metabolic pathways such as glycolysis, citrate cycle and pyruvate metabolism. This transcriptional profile was reported to be associated with differentiated intestinal cells in mammals along the crypt-villi axis [39,40]. In the present study, the entire intestine between stomach and rectum was analysed due to size limitation and the increased expression of metabolic and digestive pathways may reflect the relative proportion of different cell types, suggesting that the intestine of parr at 2850°dpf contained a higher degree of differentiated cells compared to fry at 1990°dpf. Similarly, analysis of the intestinal transcriptome also revealed a higher expression of genes involved in RNA processing and protein synthesis that implied an increased level of proliferating cells in parr compared with fry. It is not counter-intuitive to hypothesize an increase of both proliferating and differentiated cells in more mature intestine, as high cell proliferation is a characteristic of active intestinal function.

Certainly, the 1990°dpf (~2.5 g) to 2850°dpf (~10 g) window appeared a critical time of maturation that included acquiring the capability for endogenous synthesis of phospholipids to enable formation of lipoproteins, specifically chylomicrons, for the efficient export of neutral lipids to liver and other tissues. At the transcriptional level this was reflected in increased glycerophospholipid metabolism, including up-regulation of anabolic genes involved in the pathway of PC biosynthesis. Synthesis of PC can occur via two main biosynthetic pathways, the CDP-choline and PEMT (phosphatidylethanolamine N-methyltransferase) pathways. The CDP-choline pathway can occur in all nucleated cells and is involved in producing PC from choline, ATP, CTP and diacylglycerol, catalyzed by the enzymes choline kinase, choline-phosphate cytidylyltransferase and diacylglycerol cholinephosphotransferase [44]. The PEMT pathway is quantitatively significant only in hepatocytes and involves the enzyme PEMT that catalyzes the conversion of PE to PC via three sequential methylations reactions [44]. The present study showed that in fish fed the low phospholipid diet, intestine of parr (2850°dpf) had higher expression of at least two genes of the CDP-choline pathway including choline-phosphate cytidylyltransferase and diacylglycerol cholinephosphotransferase as well as higher PEMT expression compared to that of fry (1990°dpf). As diet was the same, these differences in gene expression reflect changes related to development. Notably, the up-regulation of glycerophospholipid metabolism genes corresponded with the reduction of cellular lipids (steatosis) observed in enterocytes of fry at 1990°dpf [11]. These data supported the hypothesis that intestinal steatosis observed in some fry after feeding a low phospholipid diet may be associated with enterocyte phospholipid biosynthetic pathways being incapable of producing sufficient phospholipid to support lipoprotein formation necessary for the export of dietary lipid from the gut [3]. In the present study only 20% of fish (at 1990°dpf) showed intestinal steatosis increasing the probability that “healthy” fish were included in the analysis. Therefore, the above hypothesis should be tested and confirmed in earlier life stages when more fish might be affected, or by specifically selecting only fish showing the pathology although this might be challenging.

The phospholipid feeding trial demonstrated that, in addition to a quantitative requirement for phospholipid, the relative composition of different phospholipid classes (e.g. PC, PE or PI) was also important. There is evidence to support PC being the main limiting factor driving the requirement for dietary intact phospholipid. It was shown both in salmon and rainbow trout (*Onchorhynchus mykiss*) that soybean lecithin containing equal amounts of PC, PE and PI [45] resulted in inferior growth, enzymatic activity and chylomicron concentration compared to other phospholipid sources such as egg-yolk lecithin or krill oil that are rich in PC [9]. Growth was improved in Caspian brown trout (*Salmo trutta caspius*) when soybean oil was replaced with semi-purified PC, which may reflect the importance of PC compared to other phospholipid classes although it could simply be a general phospholipid effect [8]. However, PC is of critical importance as it was shown in mammalian systems that it is the main factor essential for the formation and secretion of lipoproteins [44]. Accordingly, in rainbow trout fry, PC-rich sources of phospholipid such as egg-yolk lecithin increased the level of chylomicrons compared with soybean lecithin [9]. Similarly, in salmon, phospholipid supplementation using krill oil or soybean lecithin was only equally effective in reducing steatosis if the levels of PC were similar [11]. The present study showed that, at the gene expression level, phospholipid biosynthetic pathways were not affected by dietary phospholipid level or composition in salmon fry at 1990°dpf, which could reflect that phospholipid biosynthesis pathways were not fully developed at this early life stage. It is conceivable that dietary phospholipid supplementation may affect intestinal phospholipid biosynthesis in older parr (≥ 2850°dpf) but this was not investigated in the current study.

Chylomicron formation is an essential step in lipid digestion and delivery to tissues such as the liver. Apolipoprotein A-IV, one of the most abundant apoproteins produced by the intestine as well as the most responsive to intestinal lipid absorption and chylomicron secretion, is a critical regulator of this mechanism [46,47]. The expression of the apolipoprotein A-IV (apoA-IV) is directly linked to chylomicron formation with increased levels of this protein corresponding to increased production and secretion of chylomicrons. The down-regulation of apoA-IV observed in salmon fry at 1990°dpf when fed both diets K2.6 and S3.6 was an unexpected result. Interestingly, however, ADP-ribosylation factor 1 was also down-regulated in fry fed these diets suggesting that this might reflect a specific mechanism triggered by dietary phospholipid supplementation. ADP-ribosylation factor 1 is also indirectly involved in chylomicron formation, in that it is a critical factor modulating the formation and release of lipoproteins [48]. One explanation of the results obtained with apoA-IV and ADP-ribosylation factor-1 is that by feeding fry a low phospholipid diet, emulsified lipids are absorbed into the enterocytes but not exported and increased cellular lipid in turn triggers the activation of genes involved in chylomicron formation including apoA-IV and ADP-ribosylation factor-1 but without production of chylomicrons to switch off synthesis [47]. In fry fed phospholipid with adequate PC, chylomicron synthesis occurs feeding back to reduce expression of apoA-IV and ADP-ribosylation factor-1 genes. If confirmed, this mechanism would support the phospholipid biosynthetic pathway in enterocytes as the critical factor in determining phospholipid requirement in larval and juvenile stages of fish.

A number of other metabolic pathways were also affected, to a minor extent, at the transcriptional level by dietary phospholipid. Differences were observed in steroid and fatty acid biosynthetic pathways, which were both up-regulated in response to diets supplemented with soy lecithin (S2.6 and S3.6). However, these effects on intestinal lipid metabolism were possibly a response to altered fatty acid and sterol compositions of the lipid source (vegetable vs. marine) [49] rather than directly associated with dietary phospholipid content.

The present study was the first to investigate transcriptional changes underlying intestinal development in a critical developmental phase from 1990°dpf (fry) to 2850°dpf (parr), when salmon cease to require dietary intact phospholipid and acquire the capability for endogenous synthesis in the gut. The study demonstrated that the intestinal transcriptome of parr progressed towards a profile reflecting a higher proportion of differentiated cells likely more characteristic of mature intestine. Furthermore, our previous work confirmed that intact dietary phospholipids and particularly PC are required during early development of fry with the requirement likely associated with the inability of fry to synthesize sufficient levels of phospholipid to support the transfer of dietary lipid from enterocytes to other tissues [11]. However, the present study found that, while the level and class composition of dietary phospholipids impacted on several biochemical and morphological parameters including growth during the immature fry phase, these were not mediated by major differences in intestinal transcriptome but simply by the presence of intact phospholipid/PC in the enterocyte. In other words, the role of phospholipid/PC in stimulating growth in fry was not mediated through major changes in gene expression. However, a lack of differences in gene expression should not be considered conclusive but may be result from reduced statistical power due to challenging sampling conditions associated with size of fish, developmental differences between fish and the proportions of affected and non-affected fish. Thus, future studies should aim to further elucidate the biochemical and molecular changes that underlie phospholipid requirement in fish. Understanding the mechanisms associated with lipid transport and phospholipid biosynthesis might contribute to the improved utilization of dietary lipids in adult stages.

## Supporting Information

S1 Table Comparison of results from microarray and RT-qPCR data. (DOCX)

S2 Table Detailed results of GSEA. (DOCX)

## References

[1] Coutteau P, Geurden I, Camara MR, Bergot P, Sorgeloos P. Review on the dietary effects of phospholipids in fish and crustacean larviculture. Aquaculture. 1997;155: 149–164.

[2] Cahu C, Zambonino-Infante J, Barbosa V. Effect of dietary phospholipid level and phospholipid:neutral lipid value on the development of sea bass (*Dicentrarchus labrax*) larvae fed a compound diet. Br J Nutr. 2003;90: 21–28. 12844371 10.1079/bjn2003880

[3] Tocher DR, Bendiksen EÅ, Campbell PJ, Bell JG. The role of phospholipids in nutrition and metabolism of teleost fish. Aquaculture. 2008;280: 21–34.

[4] Daprà F, Geurden I, Corraze G, Bazin D, Zambonino-Infante J, Fontagné-Dicharry S. Physiological and molecular responses to dietary phospholipids vary between fry and early juvenile stages of rainbow trout (*Oncorhynchus mykiss*). Aquaculture. 2011;319: 377–384.

[5] Cahu C, Zambonino Infante J, Takeuchi T. Nutritional components affecting skeletal development in fish larvae. Aquaculture. 2003;227: 245–258.

[6] Rinchard J, Czesny S, Dabrowski K. Influence of lipid class and fatty acid deficiency on survival, growth, and fatty acid composition in rainbow trout juveniles. Aquaculture. 2007;264: 363–371.

[7] Geurden I, Marion D, Charlon N, Coutteau P, Bergot P. Comparison of different soybean phospholipidic fractions as dietary supplements for common carp, *Cyprinus carpio*, larvae. Aquaculture. 1998;161: 225–235.

[8] Kenari AA, Sotoudeh E, Rezaei MH. Dietary soybean phosphatidylcholine affects growth performance and lipolytic enzyme activity in Caspian brown trout (*Salmo trutta Caspius*) alevin. Aquacult Res. 2011;42: 655–663.

[9] Azarm HM, Kenari AA, Hedayati M. Effect of dietary phospholipid sources and levels on growth performance, enzymes activity, cholecystokinin and lipoprotein fractions of rainbow trout (*Oncorhynchus mykiss*) fry. Aquacult Res. 2013;44: 634–644.

[10] Poston HA. Effect of body size on growth, survival, and chemical composition of Atlantic salmon fed soy lecithin and choline. 1990;52: 226–230.

[11] Taylor JF, Martinez-Rubio L, del Pozo J, Walton JM, Tinch AE, Migaud H, et al Influence of dietary phospholipid on early development and performance of Atlantic salmon (*Salmo salar*). Aquaculture. 2015;448: 262–272.

[12] Poston HA. Response of Atlantic salmon fry to feed-grade lecithin and choline. 1991;53: 224–228.

[13] Fontagné S, Geurden I, Escaffre A, Bergot P. Histological changes induced by dietary phospholipids in intestine and liver of common carp (*Cyprinus carpio* L.) larvae. Aquaculture. 1998;161: 213–223.

[14] Geurden I, Bergot P, Schwarz L, Sorgeloos P. Relationship between dietary phospholipid classes and neutral lipid absorption in newly-weaned turbot, shape *Scophthalmus maximus* . Fish Physiol Biochem. 1998;19: 217–228.

[15] Olsen RE, Myklebust R, Kaino T, Ringo E. Lipid digestibility and ultrastructural changes in the enterocytes of Arctic char (*Salvelinus alpinus* L.) fed linseed oil and soybean lecithin. Fish Physiol Biochem. 1999;21: 35–44.

[16] Liu J, Caballero MJ, Izquierdo M, El-Sayed Ali T, Hernandez-Cruz CM, Valencia A, et al Necessity of dietary lecithin and eicosapentaenoic acid for growth, survival, stress resistance and lipoprotein formation in gilthead sea bream *Sparus aurata*. 2002;68: 1165–1172.

[17] Salhi M, Hernández-Cruz CM, Bessonart M, Izquierdo MS, Fernández-Palacios H. Effect of different dietary polar lipid levels and different n−3 HUFA content in polar lipids on gut and liver histological structure of gilthead seabream (*Sparus aurata*) larvae. Aquaculture. 1999;179: 253–263.

[18] Geurden I, Radünz-Neto J, Bergot P. Essentiality of dietary phospholipids for carp (*Cyprinus carpio* L.) larvae. Aquaculture. 1995;131: 303–314.

[19] Geurden I, Bergot P, Van Ryckeghem K, Sorgeloos P. Phospholipid composition of common carp (*Cyprinus carpio*) larvae starved or fed different phospholipid classes. Aquaculture. 1999;171: 93–107.

[20] National Research Council (NRC). Nutrient Requirements of Fish and Shrimp. Washington, DC.: National Academy Press; 2011.

[21] Tacchi L, Bron JE, Taggart JB, Secombes CJ, Bickerdike R, Adler MA, et al Multiple tissue transcriptomic responses to *Piscirickettsia salmonis* in Atlantic salmon (*Salmo salar*). Physiol Genomics. 2011;43: 1241–1254. 10.1152/physiolgenomics.00086.2011 21878610

[22] Bicskei B, Bron J, Glover K, Taggart J. A comparison of gene transcription profiles of domesticated and wild Atlantic salmon (*Salmo salar* L.) at early life stages, reared under controlled conditions. BMC Genomics. 2014;15: 884 10.1186/1471-2164-15-884 25301270 PMC4210632

[23] Tacchi L, Secombes C, Bickerdike R, Adler M, Venegas C, Takle H, et al Transcriptomic and physiological responses to fishmeal substitution with plant proteins in formulated feed in farmed Atlantic salmon (*Salmo salar*). BMC Genomics. 2012;13: 363 10.1186/1471-2164-13-363 22853566 PMC3526460

[24] Martinez-Rubio L, Morais S, Evensen Ã, Wadsworth S, Ruohonen K, Vecino JLG, et al Functional feeds reduce heart inflammation and pathology in Atlantic salmon (*Salmo salar* L.) following experimental challenge with Atlantic salmon reovirus (ASRV). PLoS ONE. 2012;7: e40266 10.1371/journal.pone.0040266 23226193 PMC3511526

[25] Morais S, Taggart J, Guy D, Bell J, Tocher D. Hepatic transcriptome analysis of inter-family variability in flesh n-3 long-chain polyunsaturated fatty acid content in Atlantic salmon. BMC Genomics. 2012;13: 410 10.1186/1471-2164-13-410 22905698 PMC3463449

[26] Morais S, Silva T, Cordeiro O, Rodrigues P, Guy DR, Bron JE, et al Effects of genotype and dietary fish oil replacement with vegetable oil on the intestinal transcriptome and proteome of Atlantic salmon (*Salmo salar*). BMC Genomics. 2012;13: 448 22943471 10.1186/1471-2164-13-448 PMC3460786

[27] De Santis C, Bartie KL, Olsen RE, Taggart JB, Tocher DR. Nutrigenomic profiling of transcriptional processes affected in liver and distal intestine in response to a soybean meal-induced nutritional stress in Atlantic salmon (*Salmo salar*). 2015;15: 1–11. 10.1016/j.cbd.2015.04.001 25916579

[28] R Core Team. R: A language and environment for statistical computing. R Foundation for Statistical Computing, Vienna, Austria http://www.R-project.org/. 2013.

[29] Gentleman R, Carey V, Bates D, Bolstad B, Dettling M, Dudoit S, et al Bioconductor: open software development for computational biology and bioinformatics. Genome Biol. 2004;5: R80 15461798 10.1186/gb-2004-5-10-r80 PMC545600

[30] Smyth GK. Linear models and empirical bayes methods for assessing differential expression in microarray experiments. Stat Appl Genet Mol Biol. 2004;3: Article3. 10.2202/1544-6115.1027 16646809

[31] Benjamini Y, Hochberg Y. Controlling the false discovery rate: A practical and powerful approach to multiple testing. 1995;57: pp. 289–300.

[32] Moriya Y, Itoh M, Okuda S, Yoshizawa AC, Kanehisa M. KAAS: an automatic genome annotation and pathway reconstruction server. Nucleic Acids Res. 2007;35: W182–5. 17526522 10.1093/nar/gkm321 PMC1933193

[33] Altschul SF, Gish W, Miller W, Myers EW, Lipman DJ. Basic local alignment search tool. J Mol Biol. 1990;215: 403–410. 2231712 10.1016/S0022-2836(05)80360-2

[34] Wickham H. ggplot2: elegant graphics for data analysis. 2009.

[35] Luo W, Friedman MS, Shedden K, Hankenson KD, Woolf PJ. GAGE: generally applicable gene set enrichment for pathway analysis. BMC Bioinformatics. 2009;10: 161 10.1186/1471-2105-10-161 19473525 PMC2696452

[36] Carmona-Antoñanzas G, Taylor JF, Martinez-Rubio L, Tocher DR. Molecular mechanism of dietary phospholipid requirement of early life stages of Atlantic salmon (*Salmo salar* L.). Biochim Biophys Acta—Mol Cell Biol Lipids. 2015;1851: 1428–1441. 10.1016/j.bbalip.2015.08.006 26303578

[37] Warnes GR, Bolker B, Bonebakker L, Gentleman R, Liaw WHA, Lumley T, et al gplots: Various R programming tools for plotting data; 2013.

[38] Noah TK, Donahue B, Shroyer NF. Intestinal development and differentiation. Exp Cell Res. 2011;317: 2702–2710. 10.1016/j.yexcr.2011.09.006 21978911 PMC3210330

[39] Mariadason JM, Nicholas C, L’Italien KE, Zhuang M, Smartt HJM, Heerdt BG, et al Gene expression profiling of intestinal epithelial cell maturation along the crypt-villus axis. Gastroenterology. 2005;128: 1081–1088. 15825089 10.1053/j.gastro.2005.01.054

[40] Chang J, Chance MR, Nicholas C, Ahmed N, Guilmeau S, Flandez M, et al Proteomic changes during intestinal cell maturation in vivo. 2008;71: 530–546. 10.1016/j.jprot.2008.08.003 PMC2655360 18824147

[41] Grosell M, Farrell AP, Brauner CJ. The multifunctional gut of fish. London, UK: Elsevier; 2011.

[42] García Hernández M.P., Lozano MT, Elbal MT, Agulleiro B. Development of the digestive tract of sea bass (*Dicentrarchus labrax* L). Light and electron microscopic studies. Anat Embryol (Berl). 2001;204(1): 39–57. 11506432 10.1007/s004290100173

[43] Sahlmann C., Gu J., Kortner T.M., Lein I., Krogdahl A., Bakke A.M. Ontogeny of the digestive system of Atlantic salmon (*Salmo salar* L.) and effects of soybean meal from start feeding. PLoS ONE 10(4): e0124179 10.1371/journal.pone.0124179 25923375 PMC4414279

[44] Cole LK, Vance JE, Vance DE. Phosphatidylcholine biosynthesis and lipoprotein metabolism. 2012;1821: 754–761. 10.1016/j.bbalip.2011.09.009 21979151

[45] Palacios L, Wang T. Egg-yolk lipid fractionation and lecithin characterization. J Am Oil Chem Soc. 2005;82: 571–578.

[46] Kalogeris TJ, Rodriguez M, Tso P. Control of Synthesis and Secretion of Intestinal Apolipoprotein A-IV by Lipid. J Nutr. 1997;127: 537S–543S. 9082042 10.1093/jn/127.3.537S

[47] Kohan AB, Wang F, Li X, Bradshaw S, Yang Q, Caldwell JL, et al Apolipoprotein A-IV regulates chylomicron metabolism mechanism and function. 2011;302: G628–G636. 10.1152/ajpgi.00225.2011 PMC3311309 22207575

[48] Asp L, Magnusson B, Rutberg M, Li L, Borén J, Olofsson S. Role of ADP Ribosylation Factor 1 in the Assembly and Secretion of ApoB-100–Containing Lipoproteins. Arterioscler Thromb Vasc Biol. 2005;25: 566–570. 15618550 10.1161/01.ATV.0000154135.21689.47

[49] Morais S, Silva T, Cordeiro O, Rodrigues P, Guy D, Bron J, et al Effects of genotype and dietary fish oil replacement with vegetable oil on the intestinal transcriptome and proteome of Atlantic salmon (*Salmo salar*). BMC Genomics. 2012;13: 448 22943471 10.1186/1471-2164-13-448 PMC3460786

