# Supplementary material for: Influence of Development and Dietary Phospholipid Content and Composition on Intestinal Transcriptome of Atlantic Salmon (Salmo salar)
Source: PLoS One. 2015 Oct 21;10(10):e0140964. doi: 10.1371/journal.pone.0140964 (PMC4619195; doi:10.1371/journal.pone.0140964)
Supplement: S1 Table — (DOCX) [file pone.0140964.s001.docx]

**Supplementary Table 1**. Comparison of results from microarray and RT-qPCR data.

|  |  |  | Microarray | | RT-qPCR^1^ | |
| --- | --- | --- | --- | --- | --- | --- |
| **Probename** | **KEGG ID** | **Name** | **Log FC** | ***p* Value** | **Log FC** | ***p* Value** |
| Ssa#S31983331 | K00999 | CDP-diacylglycerol--inositol 3-phosphatidyltransferase | 1.08 | 0.380 | 1.61 | 0.000 |
| Ssa#S30285614 | K00981 | phosphatidate cytidylyltransferase | -1.02 | 0.835 | -1.20 | 0.241 |
| Ssa#TC105286 | K13644 | choline/ethanolamine | -1.40 | 0.000 | 1.41 | 0.000 |
| Ssa#S30292122 | K00994 | diacylglycerol cholinephosphotransferase | 1.57 | 0.002 | 1.92 | 0.000 |
| Ssa#STIR15718 | K08744 | cardiolipin synthase | 1.99 | 0.000 | 2.27 | 0.000 |
| Ssa#S31973918 | K00894 | ethanolamine kinase | -1.20 | 0.110 | 1.38 | 0.026 |
| Ssa#S32011020 | K15728 | phosphatidate phosphatase LPIN | -1.80 | 0.000 | -1.49 | 0.104 |
| Ssa#S35476642 | K08536 | liver X receptor alpha | 1.11 | 0.174 | 1.20 | 0.079 |
| Ssa#S35667088 | K00551 | phosphatidylethanolamine N-methyltransferase | 1.50 | 0.000 | 1.77 | 0.002 |
| Ssa#S31995831 | K00995 | CDP-diacylglycerol--glycerol-3-phosphate 3-phosphatidyltransferase | 1.86 | 0.000 | 1.55 | 0.000 |
| Ssa#DW469378 | K01613 | phosphatidylserine decarboxylase | 1.09 | 0.135 | 1.69 | 0.002 |
| Ssa#TC107590 | K05857 | phosphatidylinositol phospholipase C, delta | 1.79 | 0.000 | 1.36 | 0.005 |

^1^ RT-qPCR data is published separately.

Log FC is log transformed fold change, where red and green denote up and down-regulated respectively.

*p* value is the FDR-uncorrected *p* value for microarray and calculated by one-way ANOVA for RT-qPCR.
