# Supplementary material for: Influence of Development and Dietary Phospholipid Content and Composition on Intestinal Transcriptome of Atlantic Salmon (Salmo salar)
Source: PLoS One. 2015 Oct 21;10(10):e0140964. doi: 10.1371/journal.pone.0140964 (PMC4619195; doi:10.1371/journal.pone.0140964)
Supplement: S2 Table — (DOCX) [file pone.0140964.s002.docx]

**Supplementary Table 2**. Detailed results of GSEA.

| **Gene Set** | **Biological Process** | **Class** | **Number of genes** | **Fold Change** | ***q* Value** |
| --- | --- | --- | --- | --- | --- |
| ***S1.5 10g vs S1.5 1g*** |  |  |  |  |  |
| Lysosome | Cellular Processes | Transport and catabolism | 89 | -4.36 | < 0.0001 |
| Carbon fixation in photosynthetic organisms | Metabolism | Energy metabolism | 15 | 3.44 | < 0.0001 |
| Ribosome | GIP | Translation | 120 | 3.08 | < 0.0001 |
| Collecting duct acid secretion | Organismal Systems | Excretory system | 15 | -2.71 | < 0.0001 |
| Glycolysis / Gluconeogenesis | Metabolism | Carbohydrate metabolism | 33 | 2.47 | < 0.0001 |
| Phagosome | Cellular Processes | Transport and catabolism | 79 | -2.41 | < 0.0001 |
| Carbohydrate digestion and absorption | Organismal Systems | Digestive system | 16 | 2.40 | < 0.0001 |
| Synaptic vesicle cycle | Organismal Systems | Nervous system | 38 | -2.11 | < 0.0001 |
| Citrate cycle (TCA cycle) | Metabolism | Carbohydrate metabolism | 23 | 2.13 | < 0.0001 |
| Cytokine-cytokine receptor interaction | EIP | Signaling molecules and interaction | 117 | -2.03 | < 0.0001 |
| Antigen processing and presentation | Organismal Systems | Immune system | 34 | -2.06 | < 0.0001 |
| Other glycan degradation | Metabolism | Glycan biosynthesis and metabolism | 13 | -2.09 | < 0.0001 |
| Glycosaminoglycan degradation | Metabolism | Glycan biosynthesis and metabolism | 13 | -2.08 | < 0.0001 |
| Histidine metabolism | Metabolism | Amino acid metabolism | 15 | 1.96 | 0.0001 |
| Propanoate metabolism | Metabolism | Carbohydrate metabolism | 22 | 1.92 | 0.0001 |
| Glycine, serine and threonine metabolism | Metabolism | Amino acid metabolism | 31 | 1.86 | 0.0001 |
| Aminoacyl-tRNA biosynthesis | GIP | Translation | 28 | 1.85 | 0.0001 |
| Pyruvate metabolism | Metabolism | Carbohydrate metabolism | 26 | 1.80 | 0.0002 |
| Intestinal immune network for IgA production | Organismal Systems | Immune system | 28 | -1.76 | 0.0003 |
| Starch and sucrose metabolism | Metabolism | Carbohydrate metabolism | 23 | 1.73 | 0.0003 |
| Insulin signaling pathway | Organismal Systems | Endocrine system | 69 | 1.68 | 0.0004 |
| Ribosome biogenesis in eukaryotes | GIP | Translation | 64 | 1.67 | 0.0004 |
| Spliceosome | GIP | Transcription | 111 | 1.62 | 0.0005 |
| Aldosterone-regulated sodium reabsorption | Organismal Systems | Excretory system | 17 | 1.67 | 0.0005 |
| Chemokine signaling pathway | Organismal Systems | Immune system | 100 | -1.65 | 0.0005 |
| Fat digestion and absorption | Organismal Systems | Digestive system | 19 | 1.63 | 0.0007 |
| RNA transport | GIP | Translation | 116 | 1.53 | 0.0011 |
| Methane metabolism | Metabolism | Energy metabolism | 14 | 1.57 | 0.0012 |
| Steroid biosynthesis | Metabolism | Lipid metabolism | 14 | 1.45 | 0.0035 |
| Pentose phosphate pathway | Metabolism | Carbohydrate metabolism | 18 | 1.39 | 0.0041 |
| Steroid hormone biosynthesis | Metabolism | Lipid metabolism | 24 | 1.37 | 0.0044 |
| Protein processing in endoplasmic reticulum | GIP | Folding, sorting and degradation | 121 | 1.32 | 0.0058 |
| Glyoxylate and dicarboxylate metabolism | Metabolism | Carbohydrate metabolism | 20 | 1.27 | 0.0100 |
| Meiosis - yeast | Cellular Processes | Cell growth and death | 44 | 1.24 | 0.0103 |
| Glycosphingolipid biosynthesis - ganglio series | Metabolism | Glycan biosynthesis and metabolism | 11 | -1.34 | 0.0126 |
| Proximal tubule bicarbonate reclamation | Organismal Systems | Excretory system | 12 | 1.18 | 0.0212 |
| One carbon pool by folate | Metabolism | Metabolism of cofactors and vitamins | 13 | 1.17 | 0.0214 |
| mRNA surveillance pathway | GIP | Translation | 54 | 1.11 | 0.0242 |
| Oxidative phosphorylation | Metabolism | Energy metabolism | 107 | 1.09 | 0.0286 |
| Mineral absorption | Organismal Systems | Digestive system | 27 | -1.17 | 0.0318 |
| Ubiquinone and other terpenoid-quinone biosynthesis | Metabolism | Metabolism of cofactors and vitamins | 10 | 1.10 | 0.0336 |
| Tryptophan metabolism | Metabolism | Amino acid metabolism | 30 | 1.05 | 0.0349 |
| Terpenoid backbone biosynthesis | Metabolism | Metabolism of terpenoids and polyketides | 17 | 1.05 | 0.0366 |
| Vitamin digestion and absorption | Organismal Systems | Digestive system | 16 | -1.15 | 0.0409 |
| Focal adhesion | Cellular Processes | Cell communication | 122 | -1.10 | 0.0443 |
| Leukocyte transendothelial migration | Organismal Systems | Immune system | 65 | -1.08 | 0.0498 |
| ***S2.6 1g vs S1.5 1g*** |  |  |  |  |  |
| Fatty acid elongation | Metabolism | Lipid metabolism | 18 | 1.46 | 0.0374 |
| Steroid biosynthesis | Metabolism | Lipid metabolism | 14 | 1.51 | 0.0374 |
| ***S3.6 1g vs S1.5 1g*** |  |  |  |  |  |
| DNA replication | GIP | Replication and repair | 33 | 1.80 | 0.0028 |
| ***K2.6 1g vs S1.5 1g*** |  |  |  |  |  |
| Valine, leucine and isoleucine degradation | Metabolism | Amino acid metabolism | 38 | -1.39 | 0.0353 |
| PPAR signaling pathway | Organismal Systems | Endocrine system | 45 | -1.43 | 0.0353 |
| Lysosome | Cellular Processes | Transport and catabolism | 89 | -1.32 | 0.0412 |
| Peroxisome | Cellular Processes | Transport and catabolism | 64 | -1.32 | 0.0412 |

*q* value is the *p* value adjusted for multiple testing corrections (Benjamini & Hochberg).

Pathways are ordered by decreasing significance.

Fold Change is the average of individual statistics and denotes how affected was the pathway.

Pathways, Biological Processes and Class are as per KEGG classification.

Number of Genes indicates the genes tested for the specific gene-set.

GIP is Genetic Information Processing

EIP is Environmental Information Processing
